# Activation of pro-oncogenic pathways in colorectal hyperplastic polyps

**DOI:** 10.1186/1471-2407-13-531

**Published:** 2013-11-08

**Authors:** Catherine Do, Claudine Bertrand, Julien Palasse, Marie-Bernadette Delisle, Elizabeth Cohen-Jonathan-Moyal, Catherine Seva

**Affiliations:** 1 INSERM UMR.1037-Cancer Research Center of Toulouse (CRCT), Université Paul Sabatier, 31052 Toulouse cedex III, Toulouse, France; 2 Service d’Anatomie Pathologique et Histologie Cytologie, CHU Rangueil and Faculté de Médecine Rangueil, Toulouse, France; 3 Institut Claudius Regaud, Toulouse, France

**Keywords:** Hyperplastic polyps, Colorectal, Progastrin, ERK, STAT3, Pro-oncogenic pathways

## Abstract

**Background:**

In contrast to sessile serrated adenomas and traditional serrated adenomas which are associated with a significant cancer risk, the role of hyperplastic polyps (HP) in colorectal carcinogenesis as well as the molecular mechanisms underlying their development remain controversial and still need to be clarified. Several reports suggest that a subset of HP may represent precursor lesions of some colorectal cancers. However, biomarkers are needed to identify the subset of HP that may have a malignant potential. The hormone precursor, progastrin (PG) has been involved in colon carcinogenesis and is known to activate pro-oncogenic pathways such as the ERK or the STAT3 pathway. We therefore analyzed PG expression and the activation of these signaling factors in HP.

**Methods:**

We retrospectively analyzed PG expression as well as the phosphorylation of ERK and STAT3 by immunohistochemistry in HP from 48 patients.

**Results:**

Mean percentages of epithelial cells positive for PG or phospho-ERK were respectively, 31% and 33% in HP and were significantly higher in these lesions compared to normal colon (3%, p = 0.0021 and 7%, p = 0.0008, respectively). We found a significant correlation between PG and phospho-ERK expression in HP with ERK activation significantly stronger in lesions with high progastrin expression (p = 0.015). In contrast, STAT3 was not significantly activated in HP compared to normal colon and we did not observe a significant correlation with PG expression.

**Conclusions:**

HP overexpressing PG that have the highest activation of the ERK pathway might reflect less latent lesions that might have a malignant potential.

## Background

Among colorectal polyps, the serrated adenomas represent a heterogeneous group of lesions including, sessile serrated adenomas (SSA) and traditional serrated adenomas (TSA). They are associated with a significant cancer risk and represent neoplastic precursor lesion of serrated adenocarcinomas [1,2]. Both SSA and TSA have a high frequency of DNA methylation. However, SSA have been linked to adenocarcinomas with microsatellite instability (MSI) with a positive immunostaining for Cytokeratin 7 (CK7) and mostly localized in the proximal colon. In contrast TSA are essentially microsatellite stable or show low level of MSI and lead to serrated adenocarcinomas in the distal colon with a positive immunostaining for CK7 and CK20. In addition, SSA are frequently BRAF-mutated whereas TSA show a high frequency of K-ras mutations [3-5].

The hyperplastic polyps (HP) are the most frequently occurring lesions in the colon with prevalence in western populations of 10% to 35% [6]. HP are usually considered as innocuous lesions with no malignant potential. However, large HP (size > 10 mm) and the presence of multiple HP (number > 5) in hyperplastic polyposis syndrome have been clearly associated with colorectal adenomas or adenocarcinoma [7-10]. However, some authors have proposed that a subset of HP may be associated to an increased risk to develop adenomas. Huang et al. [11] found that patients with HP on initial colonoscopic examination have an increased incidence of colorectal adenomas on follow-up colonoscopy. In addition, we recently published a retrospective study in which 41% of patients, without history of colorectal pathology, presenting initial true HP (<10 mm) with no SSA or TSA features, subsequently developed adenomas after resection of these HP [12]. Interestingly HP from patients who developed adenomas overexpressed the prohormone progastrin (PG) which is recognized as a growth factor, playing an important role in colon carcinogenesis [12].

PG is the precursor of the amidated gastrin. This hormone is mainly produced by antral G cells of the stomach and is known as a potent stimulant of gastric acid secretion [13]. In colorectal cancers, gastrin gene expression is up-regulated [14]. However, in these tumors, gastrin is incompletely maturated and gastrin precursors, particularly PG, are mainly secreted. High concentrations of PG are found in colon tumors and in blood of patients with colorectal cancer. PG is also expressed in adenomatous polyps [15-17]. In contrast, this hormone precursor is absent from the healthy intestinal epithelium. The proliferative effects of PG on normal and cancerous colorectal cells *in vitro* and *in vivo* have been clearly established [18-25]. In addition transgenic mice overexpressing progastrin present an increased proliferative index in colonic mucosa. They also have an increased risk of developing preneoplastic lesions in colonic epithelium [22]. These effects are mediated through the activation of signaling pathways such as the extracellular signal-regulated kinase (ERK) and the signal transducer and activator of transcription 3 (STAT3) pathways [20]. These pathways that transduce extracellular signals to the nucleus and regulate gene transcription are known to be activated in many human cancers, including colorectal cancer [20,26-30]. They have been shown to regulate cell functions involved in carcinogenesis, such as cell proliferation, survival or migration. In the present study, to better characterize at the molecular level the subset of HP that may be associated with a risk to develop colonic neoplasm, we assessed the activation of the ERK and STAT3 pathways in HP and we analyzed the correlation with PG expression.

## Methods

### Patients and data collection

We examined 48 cases of HP (from 48 different patients) diagnosed in the pathology department of Rangueil Hospital (Toulouse, France) in 2008. We excluded patients with a history of familial adenomatous polyposis or hyperplastic polyposis and HP which display criteria of sessile serrated adenomas according to Torlakovic [9,31]. All polyps measured less than 1 cm with an average diameter of 3 mm. For comparison, we also selected 12 normal colonic tissue specimens from resected non-complicated diverticular disease and 15 adenomas (10 low, and 5 high grade dysplasia adenomas). Clinical data (age, gender, site, size, number of HP at diagnosis, history of colorectal adenoma, or adenocarcinoma and the presence of synchronous adenoma or adenocarcinoma were collected for all 48 patients (Table 1). Approval of an institutional research ethics committee (Medical University of Toulouse) was obtained in accordance with the precepts of the Helsinki Declaration.

**Table 1:** Clinical and histological features of hyperplastic polyps

**Variables**	**Total of HP**	**Progastrin staining in HP epithelial cells**			
	**N = 48**	**No/Low expression**	**Moderate expression**	**High expression**	**p-value**
		**N = 21**	**N = 12**	**N = 15**	
**Age,** mean (SD)	65 y.o.(12)	61 y.o.(14)	68 y.o (10)	68 y.o (8)	0.2613
Median [min-max]	66 y.o. [30–89]	65 y.o. [30–80]	70 y.o. [51–89]	66 y.o. [53–79]	
**Sex,** % (95% CI)					0.019
Female	44% [29%-59%]	33% [15%-57%]	25% [5%-57%]	73% [45%-92%]	
Male	56% [41%-71%]	67% [43%-85%]	75% [43%-95%]	27% [8%-55%]	
**History of adenoma or adenocarcinoma,** % (95% CI)	29% [17%-44%]	33% [15%-57%]	42% [15%-72%]	13% [2%-40%]	0.280a
**Synchronous Adenoma or adenocarcinoma,** % (95% CI)	48% [33%-63%]	48% [26%-70%]	33% [10%-65%]	60% [32%-84%]	0.387
**Localisation,** % (95% CI)					0.154a
Proximal colon	27% [15%-42%]	19% [5%-42%]	17% [2%-48%]	47% [21%-73%]	
Distal colon	73% [58%-85%]	81% [58%-95%]	83% [52%-98%]	53% [27%-79%]	
**HP histologic features**					0.784a
Goblet-cell rich HP	83% [70%-93%]	81% [58%-95%]	92% [62%-100%]	80% [52%-96%]	
Microvesicular HP	17% [7%-30%]	19% [5%-42%]	8% [0%-38%]	20% [4%-48%]	

Progastrin expression was analyzed as a 3 classes variable: no/low expression, moderate expression and high expression as detailed in “Methods”. Chi 2 tests (for categorical variables) and Kruskal-Wallis test (for continuous variables) were performed to compare clinical and immune-histological features between progastrin expression groups. 95% CI: Binomial exact 95- confidence interval was calculated for each percentage, if no other mention. SD: standard deviation a: Fischer exact test.

### Immunohistochemistry

For immunohistochemistry on the formaldehyde-fixed, paraffin embedded tissues, antigen retrieval was performed on dewaxed sections by water-bath heating slides in 10 mM Tris-EDTA buffer (pH9) (Cliniscience, Nanterre, France). After peroxidase and serum blocking, primary antibodies was applied overnight. We then, used the Dako Envision + System-HRP according to manufacturer protocol (Cliniscience). Specific primary polyclonal antibody against PG used for immunohistochemistry (dilution: 1:1000) was previously characterized [12,32]. Primary monoclonal antibodies against tyrosine^705^-phospho-STAT3 (pY-STAT3) and threonin^202^/tyrosin^204^-phospho-ERK-1/2 (pERK1/2) (dilution 1:400) were provided by Cell Signaling Technology, Inc. (Danvers, MA, USA). PG antibodies were provided by the University of Melbourne, Department of Surgery (Victoria, Australia). Colonic tissue sections known to be positive for PG, p-ERK and p-STAT3 were used as positive controls. For negative controls the primary antibody was omitted. In addition, the anti-PG antibody was incubated with the immunizing peptide that abolished the staining reaction. Analysis of the whole polyp section was performed. Staining for PG (cytoplasmic), p-ERK (cytoplasmic) and p-STAT3 (nuclear) were measured by percentage of stained epithelial cells in the whole polyp. All specimens were examined in a double blinded fashion by two pathologists trained to identify the pathological features of colonic cancer. The coefficient of concordance correlation, c-rho [33] was calculated in order to determine inter-rater agreement for immunohistochemistry staining. Because the inter-rater agreement was excellent (c-rho = 0.99 for progastrin and pY-STAT3; and c-rho = 0.98 for p-ERK staining), percentages were reported as the average results between the two readers. As defined in our previous work [12], progastrin staining was also recorded as no/low, moderate or high expression. The “normality” threshold of progastrin expression (low expression) was determined using the 95th percentile of percentage of stained cells in normal colonic tissue (<10%). Moderate expression of progastrin was defined as staining in 10% to 50% of epithelial cells and high expression as staining in more than 50% of epithelial cells.

Using the 95^th^ percentile of percentage of p-ERK stained epithelial cells in normal mucosa, we defined p-ERK overexpression as staining in more than 15% of epithelial cells. Moderate expression (defined as staining in 15% to 50% of epithelial cells) was distinguished from high expression (staining in more than 50% of epithelial cells).

Using the 95^th^ percentile of percentage of pY-STAT3 stained epithelial cells in normal mucosa, we defined pY-STAT3 overexpression as staining in more than 5% of epithelial cells. High expression was defined as staining in more than 50% of epithelial cells.

### Statistical analysis

Univariate analysis was conducted to compare clinical and immunohistochemistry findings between the different study groups using the Chi^2^ test or Fisher exact test (when required) for categorical variables and the nonparametric rank tests (Wilcoxon-Mann–Whitney or Kruskal_Wallis) or Cuzick nonparametric test for trend across ordered groups for quantitative variables.

Spearman nonparametric correlation test was used to assess the correlations between the expression of progastrin and p-ERK or pY-STAT3.

All tests were two-sided and statistical significance was set at a p value of 0.05.

***p < 0.001; **0.001 < *p* < 0.01; *0.01 < *p* < 0.05. Analyses were performed using the statistical software, STATA v11 [34].

## Results

### Clinical and histological characteristics

**Figure 1:**
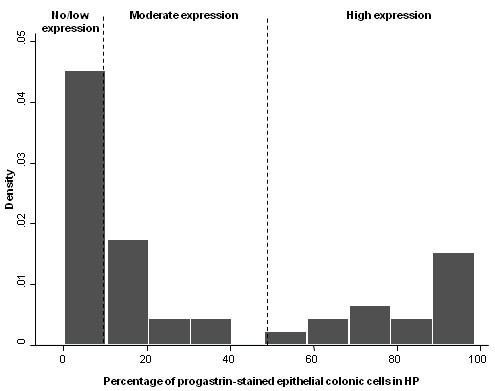
**Distribution of percentage of progastrin stained epithelial cells in HP.** No/low expression of progastrin was determined using the 95^th^ percentile of percentage of stained cells in normal colonic mucosa (< 10%). Among HP overexpressing the prohormone (> 10%), moderate expression (10%-50%) and high expression (> 50) were distinguished accordingly to the bimodal distribution of progastrin expression.

Clinical and histological features of patients and their polyps as well as PG staining in HP epithelial cells are shown in Table 1. To take into account the heterogeneity of PG staining in HP observed in Figure 1, PG expression was analyzed in Table 1 as a 3 classes variable as detailed in “Methods”. In our sample, no/low expression of progastrin was observed in 44% of the HP (95% CI: 29%-59%), moderate expression in 25% (14%-40%) and strong expression in 31% (19%-46%). Although an increased prevalence of HP with high PG expression can be observed in women, PG expression was not significantly correlated to the following variables: age, history of adenoma or carcinoma, synchronous adenoma or carcinoma, localization and HP histological features.

### Expression of progastrin in normal mucosa and colonic neoplasms

**Figure 2:**
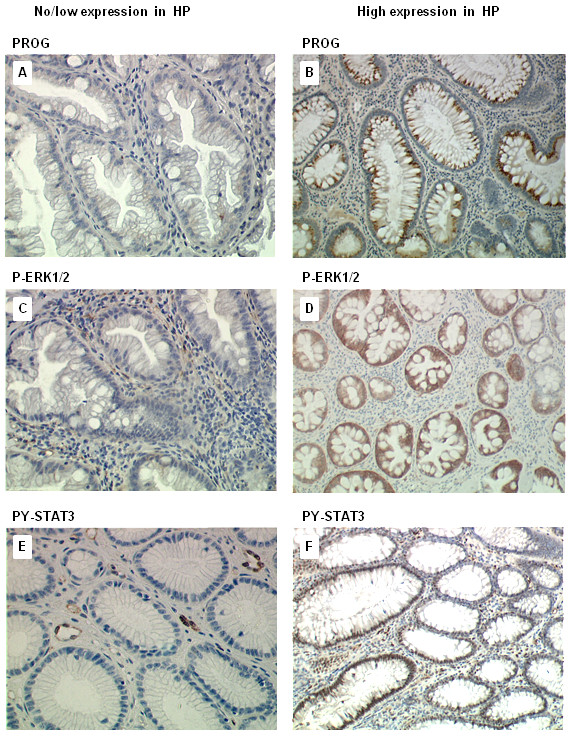
**Expression of Progastrin p-ERK1/2 and pY-STAT3 in hyperplastic polyps. (A, B)** Progastrin staining in HP: **A)** no/weak staining **B)** high progastrin staining. **(C, D)** p-ERK1/2 staining in HP: **C)** no/weak staining **D)** high p-ERK1/2 staining. **(E, F)** pY-STAT3 staining in HP: **E)** no/weak staining **F)** high pY-STAT3 staining.

**Figure 3:**
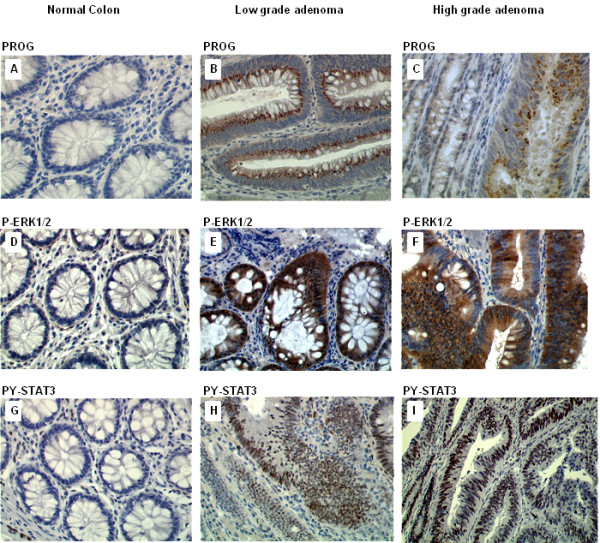
**Expression of Progastrin p-ERK1/2 and pY-STAT3 in normal colonic mucosa and adenomas. (A-C)** Progastrin staining: **A)** Negative staining in normal colon **B)** high progastrin staining in low grade and **C)** high grade adenomas. **(D-F)** p-ERK1/2 staining: **D)** Negative staining **E)** high p-ERK1/2 staining in low grade and **F)** high grade adenomas. **(G-I)** pY-STAT3 staining: **G)** Negative staining in normal colon **H)** high pY-STAT3 staining in low grade and **I)** high grade adenomas.

**Figure 4:**
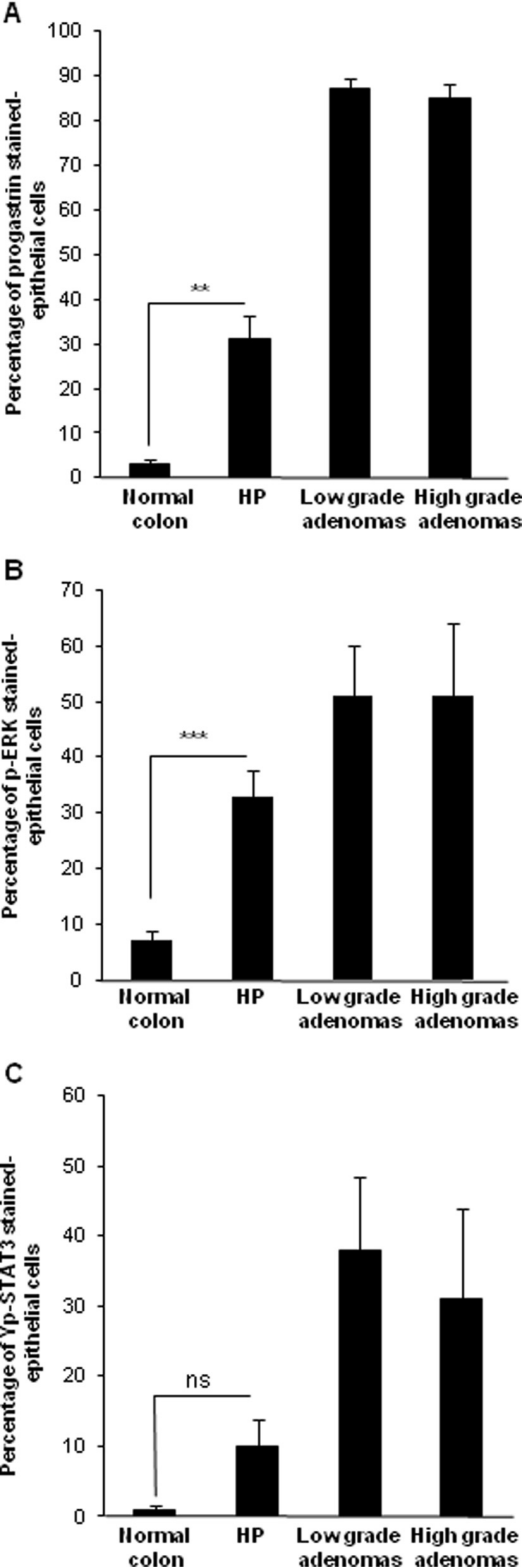
**Progastrin, p-ERK1/2, and pY-STAT3 expression in colonic tissues.** Percentage of **(A)** progastrin **(B)** p-ERK1/2 **(C)** pY-STAT3 positive cells for normal colon, Hyperplastic polyps (HP), low grade dysplasia tubular adenomas and high grade dysplasia tubular adenomas. Quantifications are presented as means ± S.E.M.

Representative pictures of PG staining, obtained with the anti-PG antibody in normal colon, HP, low grade and high grade adenomas are shown in Figures 2 and 3. The percentages of PG-positive cells in these different sample tissues are reported in Figure 4A. Mean percentage of PG positive cells observed in the 48 HP was significantly higher than in normal colon (respectively 31% and 3% p = 0.0021). In low and high grade tubular adenomas, percentages of PG positive cells were also higher compared to normal colon (respectively, 87%, p = 0.0001 and 85%, p = 0.0014). No significant difference was observed between low and high grade adenomas. HP showed an intermediate expression of PG between normal mucosa and colonic adenomas.

### The ERK pathway in normal colonic mucosa, HP and adenomas and its relationship with progastrin expression

Representative pictures of p-ERK1/2 staining in normal colon, HP, low grade and high grade adenomas are shown in Figures 2 and 3. The percentages of pERK1/2-positive cells in these different sample tissues are reported in Figure 4B. Mean percentage of p-ERK positive epithelial cells in normal colonic tissues reached 7%. In HP, the activation of ERK was significantly higher, with 33% of stained epithelial cells (p = 0.0008). In low grade and high grade adenomas, mean percentages of stained cells were similar (51%) and significantly higher than in normal mucosa (respectively, p = 0.0002 and p = 0.0105).

Interestingly, in HP, ERK activation was significantly higher in lesions with strong PG expression (53% of stained cells) as compared to no/low or moderate PG expression (respectively 29% and 22%, p = 0.015) (Table 2). As described in “methods” we also defined 3 groups for p-ERK expression. 65% of HP presented an overexpression of p-ERK, including 37% of moderate expression and 28% of high expression. Analysis of the increase in percentage across the different expression groups showed that the expression of p-ERK and PG was significantly correlated (p = 0.008) (Table 2).

**Table 2:** Expression of progastrin, p-ERK1/2 and pY-STAT3 in hyperplastic polyps

**Variables**	**Total**	**Progastrin staining in HP epithelial cells**			
	**N = 48**	**No/Low expression**	**Moderate expression**	**High expression**	**p-value**
		**N = 21**	**N = 12**	**N = 15**	
**Percentage of p-ERK1/2 stained cells,** N mean (SD)	N = 43 33% (30)	N = 18 27% (29)	N = 12 23% (22)	N = 13 53% (29)	0.015_a_
**Expression of p-ERK1/2**					0.008_b_
No/low expression	35% [21%-51%]	50% [26%-74%]	42% [15%-72%]	8% [0%-36%]	
Moderate expression	37% [23%-53%]	33% [13%-59%]	42% [15%-72%]	38% [14%-68%]	
High expression	28% [15%-44%]	17% [4%-41%]	16% [2%-48%]	54% [25%-81%]	
**Percentage of pY-STAT3 stained cells,** N mean (SD)	N = 35 10% (23)	N = 15 11% (29)	N = 7 3% (7)	N = 13 12% (23)	0.297
**Expression of pY-STAT3**					0.3116
No/low expression	77% [60%-90%]	80% [52%-96%]	86% [42%-100%]	69% [39%-91%]	
Moderate expression	14% [5%-30%]	7% [0%-32%]	14% [0%-58%]	23% [5%-54%]	
High expression	9% [2%-23%]	13% [2%-40%]	0% [0%-41%]	8% [0%-36%]	

SD: standard deviation.

(a)Cuzick test for trend across ordered groups.

(b)Spearman nonparametric correlation test was used for comparison of ordered qualitative variable.

Expressions of PG, .P-ERK and PY-STAT3 were analyzed as a 3 classes variable: no/low expression, moderate expression and high expression as detailed in “Methods”.

### The STAT3 pathway in normal colonic mucosa HP and adenomas and its relationship with progastrin expression

Representative pictures of pY-STAT3 staining in normal colon, HP, low grade and high grade adenomas are shown in Figures 2 and 3.

The percentages of pY-STAT3-positive cells in these different sample tissues are reported in Figure 4C. Mean percentage of pY-STAT3-positive cells in normal colonic mucosa was only 1%. In HP the percentage of stained cells was 10% but was not significantly higher than in normal colonic mucosa. In low grade and high grade adenomas, mean percentages of stained cells were respectively 38% and 31% and significantly higher than in normal mucosa (respectively, p = 0.0014 and p = 0.0041).

As observed for PG and P-ERK no significant difference in PY-STAT3 staining was observed between low and high grade adenomas.

3 groups for pY-STAT3 expression were defined as described in “Methods”. 23% of HP presented an overexpression of pY-STAT3, including 14% with moderate expression and 9% with high expression (Table 2). In HP, mean percentage of pY-STAT3 staining was not associated to the different classes of PG staining (p = 0.297) and no correlation between these two factors was observed (p = 0.3116).

## Discussion

In the present study, we demonstrated a significant increase in the activation of the pro-oncogenic pathway, ERK1/2, in HP as compared to normal tissue. More interestingly, we showed a significant correlation between ERK pathway activation in HP and the expression of PG that is recognized as a growth factor for colonic epithelial cells. ERK activation was significantly higher in lesions with strong PG expression. Activation of this signaling pathway by PG has been previously reported in normal colonic epithelial cells from a transgenic mouse model overexpressing PG and has been linked to an increased risk of developing preneoplastic lesions in the colonic epithelium [20]. Therefore HP overexpressing PG that have a high activation of the ERK pathway might reflect less latent lesions.

The PG gene has been previously shown to be a target of two pro-oncogenic pathways frequently activated in colorectal cancer: APC/β-catenin and K-ras [35-37]. APC deletions or β-catenin mutations have not been reported in HP and we recently published that this pathway is not activated in HP with PG overexpression [12]. Therefore, it is unlikely that this pathway is involved in the expression of PG in these lesions. In contrast, KRAS mutations have been observed in thirty-seven percent of HP [5] and might lead to the increase in PG expression and ERK activation observed in the present study. However we cannot exclude an additional mechanism leading to PG expression in HP since in our study, nearly to sixty percent of HP presented an overexpression of PG or p-ERK. In a recent publication, Bongers et al. [38] have reported the activation of the EGFR pathway in seventy percent of HP from a small cohort of 27 samples. Interestingly, EGFR ligands have been shown to be potent regulators of the progastrin gene and an EGF response element has been identified on the progastrin promoter [39,40]. Therefore the EGFR pathway activated in HP might also contribute to PG overexpression and ERK activation independently of K-ras.

Progastrin is clearly recognized as an autocrine growth factor for colorectal cancer cells and blocking PG expression has been shown to inhibit cellular growth in vitro and in vivo on tumor xenografts [23,41,42]. It is probable that an autocrine mechanism occurs in HP producing PG and leading to ERK activation. However a recent publication from Duckworth et al. [43] suggests that an indirect mechanism might be also proposed. These authors have shown that PG is capable to activate colonic fibroblasts leading to growth factors secretion that in turn stimulate colonic epithelial cells. These results therefore suggest that PG produced by HP might also activate the ERK pathway in colonic epithelial cells via a dialogue with the fibroblasts present in the stroma.

The identity of the receptor mediating the PG effects on colonic epithelial cells or fibroblasts remains an important point of debate. Several publications have shown that the receptor specific for the mature form of gastrin, the CCK-2 receptor, is not involved in the PG effect on fibroblasts or colon cancer cells [18,43,44]. In contrast the data from Jin et al. [45] suggest a role of this receptor in the proliferative effects of PG *in vivo*, although the nature of the interaction between PG and the CCK2 receptor in this study remains to be identified. Other studies have shown a role of ferric ions, Annexin A2 or glycosaminoglycans in the binding of PG to cell surface [20,46,47]. However, the cell surface protein that directly binds PG remained to be identified.

In contrast to what we observed for the ERK pathway, STAT3 activation was not significantly different between HP and normal colon. In addition we did not observe a significant correlation with PG expression. We previously demonstrated an association between STAT3 activation and PG *in vivo*, in transgenic mice overexpressing the prohormone [20]. STAT3 activation by PG might required high level of progastrin expression, as found in adenomas or adenocarcinomas.

Previously we demonstrated that PG expression in HP may predict occurrence of metachronous adenomas [12]. Including additional biomarkers might improve the specificity of such a test. P-ERK might be an interesting factor since this pro-oncogenic factor is overexpressed in a subset of PG positive HP.

## Conclusion

HP overexpressing PG that have the highest activation of the ERK pathway might reflect less latent lesions that might have a malignant potential.

### Consent

Written informed consent was obtained from the patient for the publication of this report and any accompanying images.

## Competing interests

The author(s) declare that they have no competing interests.

## Authors’ contributions

CD^1^ contributed to study conception and design, acquisition analysis and interpretation of the data, statistical analysis, drafting and revision of the manuscript. CB^1^, JP^1^ contributed to study design, acquisition and analysis of the data, and revision of the manuscript. MBD^2^ and ECJM^1^ contributed to interpretation of the data, and revision of the manuscript, CS^1^ contributed to study conception and design, data interpretation, drafting and revision of the manuscript, study supervision. All authors read and approved the final manuscript.

## Pre-publication history

The pre-publication history for this paper can be accessed here:

http://www.biomedcentral.com/1471-2407/13/531/prepub

## Aknowledgments

We thank Professor Shulkes, Melbourne University (Australia) for the specific antibody against Progastrin, and Serge Estaque for his technical assistance.
